# A Liquid-Liquid Phase Separation-Related Gene Signature as Prognostic Biomarker for Epithelial Ovarian Cancer

**DOI:** 10.3389/fonc.2021.671892

**Published:** 2021-06-08

**Authors:** Yan Qiu, Min Pan, Xuemei Chen

**Affiliations:** Department of Gynecology, Maoming People’s Hospital, Maoming, China

**Keywords:** epithelial ovarian cancer, liquid-liquid phase separation, risk stratification, prognostic biomarker, gynecologic oncology

## Abstract

**Objective:**

The aim of the present study was to construct and test a liquid-liquid phase separation (LLPS)-related gene signature as a prognostic tool for epithelial ovarian cancer (EOC).

**Materials and Methods:**

The data set GSE26712 was used to screen the differentially expressed LLPS-related genes. Functional enrichment analysis was performed to reveal the potential biological functions. GSE17260 and GSE32062 were combined as the discovery to construct an LLPS-related gene signature through a three-step analysis (univariate Cox, least absolute shrinkage and selection operator, and multivariate Cox analyses). The EOC data set from The Cancer Genome Atlas as the test set was used to test the LLPS-related gene signature.

**Results:**

The differentially expressed LLPS-related genes involved in several cancer-related pathways, such as MAPK signaling pathway, cell cycle, and DNA replication. Eleven genes were selected to construct the LLPS-related gene signature risk index as prognostic biomarker for EOC. The risk index could successfully divide patients with EOC into high- and low-risk groups. The patients in high-risk group had significantly shorter overall survival than those with in low-risk group. The LLPS-related gene signature was validated in the test set and may be an independent prognostic factor compared to routine clinical features.

**Conclusion:**

We constructed and validated an LLPS-related gene signature as a prognosis tool in EOC through integrated analysis of multiple data sets.

## Introduction

Although rapid progress was made in recent decades in identifying the genetic causes of cancers, our mechanistic understanding of these diseases remains incomplete and limits our ability to provide effective treatments. Novel concepts may be required to reveal the complex mechanisms underlying these diseases. Evidence is mounting that liquid-liquid phase separation (LLPS) (1) underlies the formation of various subcellular structures, such as membraneless bodies, heterochromatin (2), and the transport channel in the nuclear pore complex (3). LLPS has emerged as a new concept to elaborate the organization of living cells (4). Hundreds of genes (5) were considered involved in the dynamic process of LLPS in the form of protein or RNA molecules (6). The emerging evidence indicated that aberrant forms of LLPS are associated with many human diseases, including cancer (7). For instance, the FET protein family is involved in phase transitions at sites of RNA storage (8, 9) and assembles into higher-order structures by a process that is stimulated by RNA (10, 11). Notably, these functions are often impaired in human diseases, such as cancer and neurodegenerative diseases (12, 13).

Epithelial ovarian cancer (EOC) is the most lethal gynecological cancer with 46% survival five years after the diagnosis (14). A risk score system help in identifying the patients at high risk and decision-making for treatment. Thus, we hypothesized that the LLPS-related genes way be potential prognostic signature in EOC. To test our hypothesis, an LLPS-related gene signature was constructed in a discovery data set and tested in another independent data set.

## Materials and Methods

### Data Processing

**Figure 1:**
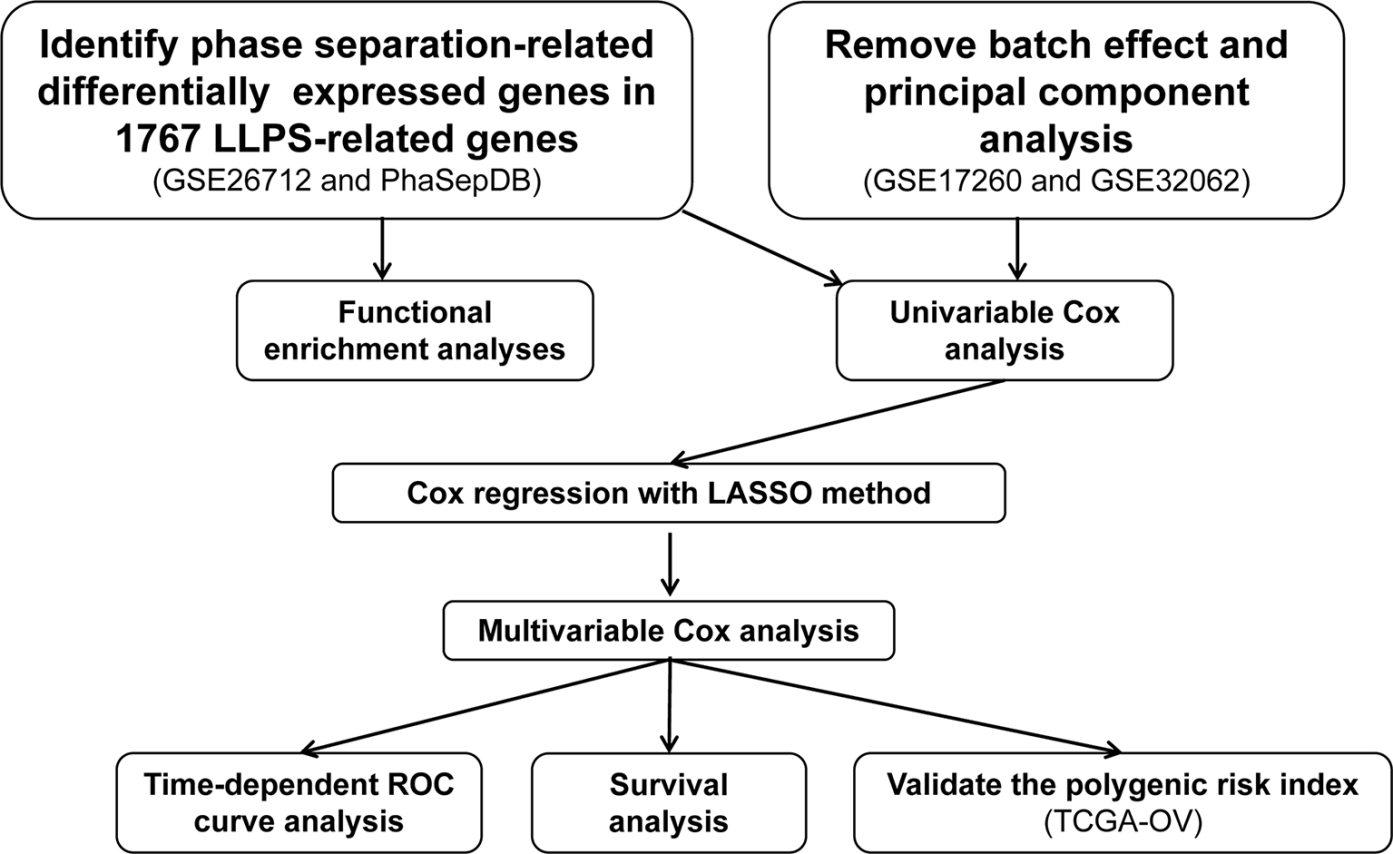
The workflow of the present study.

The LLPS-related genes were obtained from PhaSepDB (http://db.phasep.pro/) (5). Three epithelial ovarian cancer (EOC)-related processed gene expression data sets were downloaded from Gene Expression Omnibus (GEO, https://www.ncbi.nlm.nih.gov/geo/) using the “GEOquery” package (15). The data set GSE26712 (16) based on the GPL96 platform contains the gene expression profiles of 185 EOC and 10 normal ovarian surface epithelium and was used to screen the differentially expressed genes (DEGs) in EOC compared to normal ovarian surface epithelium. The data set GSE17260 (17) based on the GPL6480 platform contains the gene expression profiles of 110 EOC samples and prognosis information of the corresponding patients. The gene expression profiles of 260 EOC samples based on GPL6480 from GSE32062 (18) were also downloaded from GEO. The GSE17260 and GSE32062 were combined as the discovery set, and then the batch effects were removed using the *ComBat* function in the “sva” package (19). Principal component analysis was performed to visualize the results of removing batch effects. The discovery set was used to generate an LLPS-related gene signature in EOC. Another EOC-related data set (20), including gene expression profiles based on Affymetrix Human Genome U133 Plus 2.0 Array platform (Affymetrix; Thermo Fisher Scientific Inc., Waltham, MA, USA) and the clinical data The Cancer Genome Atlas (TCGA, https://portal.gdc.cancer.gov/) was downloaded from UCSC Xena (http://xena.ucsc.edu/) and used as the test set to validate the LLPS-related gene signature. In the above data sets, if one gene matched multiple probes, the average value of the probes was calculated as the expression of the corresponding gene. The workflow of the present was showed in **Figure 1**.

### Screen the Differentially Expressed Genes in EOC

The expression profiles of the LLPS genes were extracted from GSE26712. The DEGs between EOC and normal ovarian surface epithelium were screened using the “limma” package (21) in R. The fold changes (FCs) of individual genes were calculated, and DEGs with FCs > 1.5 and P (adjusted by false discovery rate) value < 0.05 were considered significant.

### Functional Enrichment Analysis

Functional enrichment analysis was performed to reveal the potential biological functions of the DEGs using clusterProfiler (22) package, including gene ontology (GO) and Kyoto Encyclopedia of Genes and Genomes (KEGG) pathways. P adjusted by Benjamini & Hochberg method < 0.01 and q value < 0.05 was considered significant.

### Construction of an LLPS-Related Gene Signature

In our present study, a three-step analysis was carried out to construct a robust LLPS-related gene signature for predicting prognosis in the discovery set. Firstly, univariate Cox regression analysis was performed to identify overall survival (OS)-related DEGs. A DEG with a P < 0.05 was considered a OS-related gene. Secondly, the gene expression profiles of the OS-related DEGs were subjected to least absolute shrinkage and selection operator (LASSO) Cox regression analysis using the “glmnet” package (https://CRAN.R-project.org/package=glmnet). In this analysis, the OS-related DEGs with non-zero regression coefficients were identified in 10-fold cross-validation. The relevant parameters were set to “family=“cox”,” “maxit = 1000”, and “nfolds=10”. Third, the expression profiles of the OS-related DEGs with non-zero coefficients were used to perform multivariate Cox regression analysis. The LLPS-related gene signature was constructed as the formula:

$$\text{Risk index }=\text{ Expr}{\text{gene}}_{1}*\text{Coef}{\text{gene}}_{1}+\text{ Expr}{\text{gene}}_{2}*\text{Coef}{\text{gene}}_{2}+\text{ Expr}{\text{gene}}_{3}*\text{Coef}{\text{gene}}_{3}+\ldots$$

The “Expr” represents the expression value of a gene with P < 0.05 in the multivariate Cox regression analysis. The “Coef” represents the coefficient of the corresponding gene. Each individual was assigned the LLPS-related gene signature index. The patients were divided into high- and low-risk group, and the OS between the two groups were compared.

### Validation of the LLPS-Related Gene Signature in the Test Set

As it was in the discovery set, each individual in the test set was assigned an LLPS-related gene signature index according to the above formula. Moreover, the prognostic value of the LLPS-related gene signature and the routine clinical features was compared using multivariate Cox regression analysis.

### Gene Set Enrichment Analysis

In order to reveal the biological functions of candidate genes in EOC, we performed gene set enrichment analysis (GSEA) (23, 24). We use the median expression value of each candidate gene as a threshold, and divide the EOC in TCGA into high- and low-expression groups. The canonical pathways of Kyoto Encyclopedia of Genes and Genomes gene sets derived from the Molecular Signatures Database (25) were selected as the reference gene sets. The P value adjusted by Benjamini & Hochberg method < 0.05 was set as the cut-off criteria. GSEA was performed using the clusterProfiler package and visualized using enrichplot package (https://github.com/YuLab-SMU/enrichplot).

### Statistical Analysis

In the present study, all these analyses were performed in R (version 4.0.2) (https://www.r-project.org). The DEGs were screened using unpaired t-tests provided by “limma” package. The OS was compared using Kaplan-Meier curve with log-rank method. The predictive value of the LLPS-related gene signature was evaluated by time-dependent receiver operating characteristic (tROC) curve analysis using the timeROC package (https://CRAN.R-project.org/package=timeROC). All tests were two-sided and P < 0.05, unless otherwise stated, was considered statistically significant.

## Results

### Multiple LLPS-Related Genes Aberrantly Expressed in EOC

PhaSepDB database includes 2957 eligible genes, however, a total of 1767 LLPS-related genes were found in the GSE26712, among them, 252 genes were down-regulated and 248 were up-regulated in EOC compared to the normal ovarian surface epithelium (**Figure S1A**). These DEGs showed clearly different expression patterns in EOC and normal ovarian surface epithelium (**Figure S1B**). This indicates that the abnormal state of liquid-liquid phase separation may contribute to EOC.

### Biological Functions Involved in Differentially Expressed LLPS-Related Genes

The GO enrichment analysis included cellular component (CC), biological process (BP), and molecular function (MF). The top significant (ranked by P value) 15 GO terms were showed in **Figure S2**. In CC (**Figure S2A**), the DEGs mainly involved in the composition of macromolecules and organelles, such as spliceosomal complex, ribonucleoprotein granule, and preribosome. In BP (**Figure S2B**), the DEGs significantly involved in RNA processing, such as RNA splicing, regulation of mRNA metabolic process, and RNA catabolic process. In MF (**Figure S2C**), the DEGs involve the activity of multiple enzymes, such as helicase activity, phosphatase activity, and DNA-dependent ATPase activity. The DEGs involved in several cancer-related pathways (**Figure S2D**), including MAPK signaling pathway, cell cycle, and DNA replication.

### The LLPS-Related Gene Signature in the Discovery Set

**Figure 2:**
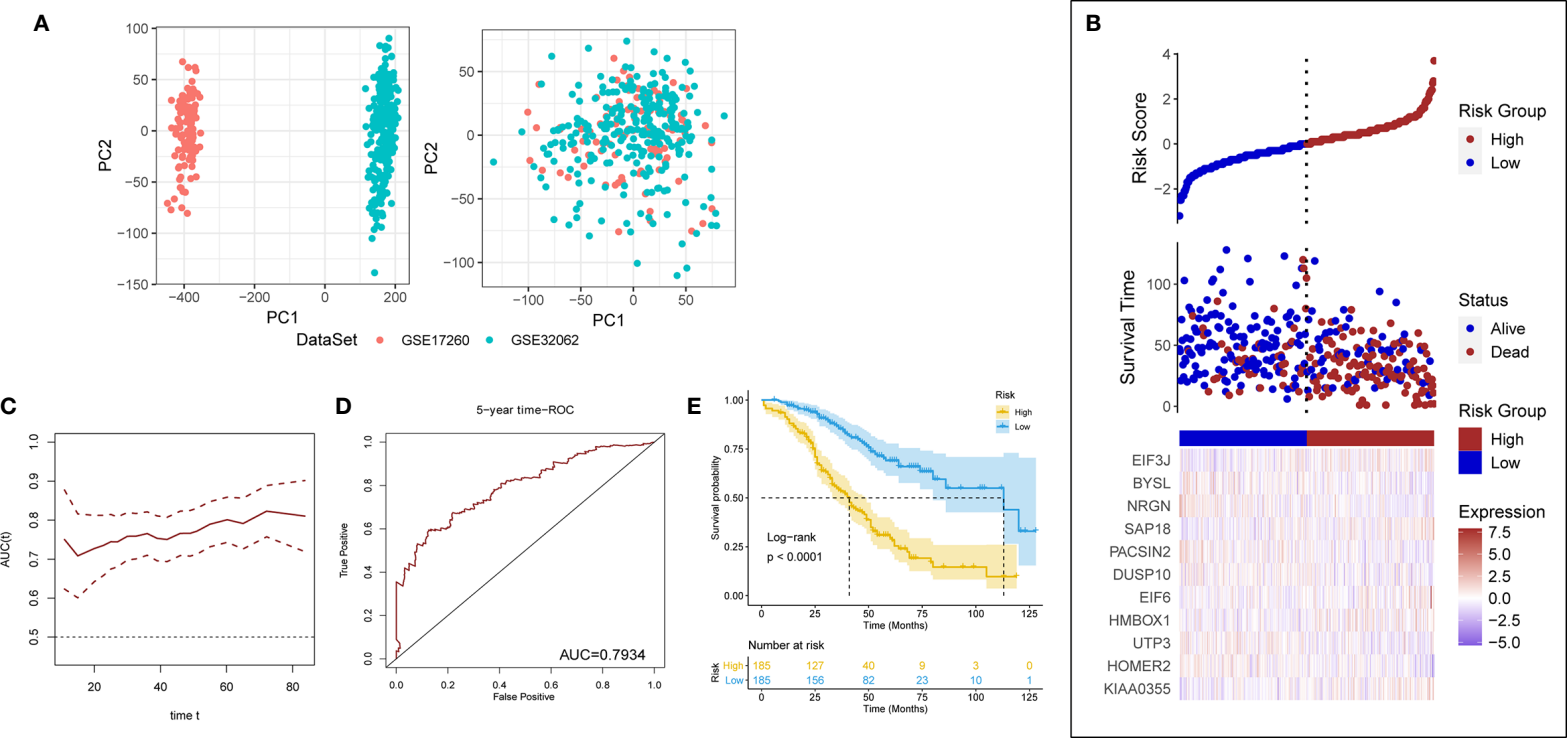
The liquid-liquid phase separation-related gene signature in the discovery set. **(A)** PCA results before (left) and after (after) removing batches between GSE17260 and GSE32062. PC, principal component. **(B)** The 11 genes of interest constitute the liquid-liquid phase separation-related gene signature. **(C)** The time receiver operating characteristic curve (ROC) analysis in the discovery set. **(D)** The five-year time ROC in the discovery set. **(E)** The patients with high risk index had significantly shorter overall survival than those with low risk index.

The results of PCA showed the GSE17260 and GSE32062 had obvious batch effects (**Figure 2A**, left), which was removed by “sva” package for the subsequent analysis (**Figure 2A**, right). Sixty differentially expressed LLPS-related genes were identified as OS-related genes by univariate Cox analysis (**Table 1**), and 32 LLPS-related genes were identified with non-zero regression coefficients by LASSO analysis (**Table 1**). Finally, 11 LLPS-related genes (EIF3J, BYSL, NRGN, SAP18, PACSIN2, DUSP10, EIF6, HMBOX1, UTP3, HOMER2, and KIAA0355) remained significantly associated with OS in multivariate Cox analysis (**Table 1**) and were selected to construct the LLPS-related gene signature risk index (RI) (**Figure 2B**). The RI was significant associated with poor prognosis (hazard ratio {HR] = 2.771, 95% confidence interval [CI] for HR = 2.272-3.379, P < 2.2e-16). The tROC curve analysis showed the predictive value of the RI was high with area under the ROC curve (AUC) = 0.7–0.8 (**Figure 2C**), and the AUC of 5-year tROC curve was 0.793 (**Figure 2D**). The patients with high RI had significantly shorter OS than those with low RI (**Figure 2E**).

**Table 1:** The overall survival-related genes and their coefficients.

Gene	Univariate Cox analysis			LASSO analysis	Multivariate Cox analysis		
	Coef	HR (95% CI for HR)	P value	Coef	Coef	HR (95% CI for HR)	P value
ANXA7	0.485	1.624 (1.139-2.316)	0.007	0.229788	0.379	1.461 (0.964-2.216)	0.074
ATP5E	0.522	1.684 (1.198-2.369)	0.003	0.161437	0.228	1.256 (0.802-1.967)	0.320
AURKA	0.187	1.205 (1.003-1.448)	0.047	0.000000			
BYSL	-0.293	0.746 (0.576-0.967)	0.027	-0.318905	-0.547	0.579 (0.405-0.827)	**0.003**
CABIN1	-0.402	0.669 (0.507-0.884)	0.005	-0.062035	-0.085	0.919 (0.621-1.360)	0.672
CCNB1	0.193	1.213 (1.000-1.472)	0.050	0.000000			
CDK7	0.328	1.388 (1.032-1.868)	0.030	0.055753	0.172	1.187 (0.818-1.723)	0.366
CEBPA	-0.204	0.816 (0.689-0.966)	0.018	-0.100185	-0.180	0.835 (0.676-1.032)	0.095
CENPA	0.145	1.156 (1.004-1.332)	0.044	0.000000			
CRNKL1	0.492	1.635 (1.233-2.168)	0.001	0.079895	0.044	1.045 (0.643-1.697)	0.860
CYBA	-0.235	0.791 (0.637-0.982)	0.034	0.000000			
DDX1	0.394	1.483 (1.121-1.963)	0.006	0.010649	-0.082	0.921 (0.586-1.448)	0.721
DDX17	-0.347	0.706 (0.551-0.906)	0.006	-0.119905	-0.153	0.858 (0.625-1.178)	0.344
DDX50	0.361	1.435 (1.062-1.938)	0.019	0.000000			
DNAJB1	0.332	1.393 (1.040-1.867)	0.026	0.180997	0.330	1.391 (0.963-2.009)	0.079
DNAJC8	0.357	1.429 (1.015-2.012)	0.041	0.000000			
DPM1	0.535	1.708 (1.290-2.262)	0.000	0.270596	0.259	1.296 (0.865-1.942)	0.209
DTYMK	0.246	1.279 (1.018-1.605)	0.034	0.000000			
DUSP10	-0.164	0.849 (0.745-0.967)	0.014	-0.089458	-0.188	0.829 (0.708-0.970)	**0.019**
EIF3J	0.494	1.638 (1.194-2.249)	0.002	0.512805	0.608	1.836 (1.166-2.892)	**0.009**
EIF6	0.370	1.448 (1.049-1.998)	0.024	0.238628	0.484	1.622 (1.120-2.348)	**0.010**
ESF1	0.291	1.337 (1.019-1.755)	0.036	0.000000			
FAM98A	0.350	1.419 (1.044-1.928)	0.025	0.012330	0.133	1.142 (0.759-1.720)	0.524
FBP1	-0.126	0.882 (0.786-0.988)	0.030	0.000000			
GTF2B	0.409	1.506 (1.029-2.204)	0.035	0.000000			
HMBOX1	0.166	1.181 (1.011-1.380)	0.036	0.149560	0.220	1.247 (1.038-1.497)	**0.018**
HOMER2	-0.168	0.846 (0.749-0.954)	0.007	-0.146708	-0.201	0.818 (0.714-0.937)	**0.004**
HP1BP3	0.307	1.359 (1.030-1.793)	0.030	0.000000			
INPP5A	0.204	1.227 (1.018-1.479)	0.032	0.034363	0.076	1.079 (0.842-1.382)	0.549
KIAA0355	0.460	1.583 (1.147-2.184)	0.005	0.318416	0.578	1.783 (1.086-2.926)	**0.022**
KIF20B	0.237	1.267 (1.042-1.540)	0.018	0.000000			
LSM14A	0.581	1.789 (1.284-2.492)	0.001	0.025548	-0.055	0.946 (0.560-1.600)	0.837
LUC7L3	0.279	1.322 (1.031-1.697)	0.028	0.000000			
LYZ	-0.106	0.900 (0.828-0.978)	0.013	0.000000			
MAP4K3	0.313	1.368 (1.058-1.768)	0.017	0.000000			
MDFIC	-0.160	0.852 (0.734-0.989)	0.036	0.000000			
NRGN	-0.252	0.777 (0.675-0.896)	0.000	-0.179244	-0.249	0.780 (0.653-0.931)	**0.006**
NSA2	0.335	1.398 (1.029-1.899)	0.032	0.000000			
OIP5	0.258	1.295 (1.072-1.564)	0.007	0.019890	0.144	1.154 (0.864-1.542)	0.331
PACSIN2	-0.393	0.675 (0.501-0.909)	0.010	-0.281363	-0.453	0.636 (0.425-0.952)	**0.028**
PI4KA	-0.301	0.740 (0.567-0.966)	0.027	-0.054323	-0.103	0.902 (0.666-1.222)	0.506
PLA2G4A	-0.187	0.829 (0.732-0.940)	0.004	-0.011256	0.006	1.006 (0.878-1.151)	0.936
PNRC2	0.608	1.836 (1.347-2.503)	0.000	0.085764	-0.064	0.938 (0.613-1.434)	0.767
PTX3	-0.097	0.907 (0.831-0.990)	0.029	-0.048561	-0.090	0.914 (0.830-1.007)	0.068
PUM2	0.407	1.502 (1.057-2.134)	0.023	0.000000			
RBBP4	0.286	1.331 (1.002-1.769)	0.048	0.000000			
RBM39	0.471	1.601 (1.080-2.374)	0.019	0.000000			
RNF34	0.371	1.449 (1.057-1.986)	0.021	0.000000			
RPL37A	0.341	1.407 (1.033-1.915)	0.030	0.000000			
RPS7	0.411	1.508 (1.130-2.011)	0.005	0.008188	0.062	1.064 (0.694-1.632)	0.774
SAE1	0.399	1.491 (1.176-1.890)	0.001	0.000000			
SAP18	0.630	1.878 (1.359-2.597)	0.000	0.478655	0.561	1.752 (1.201-2.556)	**0.004**
SNRPD2	0.499	1.648 (1.199-2.264)	0.002	0.056403	-0.001	0.999 (0.640-1.560)	0.997
SRSF7	0.510	1.666 (1.112-2.499)	0.014	0.000000			
TAGLN2	-0.245	0.783 (0.618-0.992)	0.042	0.000000			
TASP1	0.333	1.394 (1.050-1.852)	0.021	0.019793	0.182	1.200 (0.804-1.790)	0.373
TERF1	0.396	1.487 (1.059-2.087)	0.022	0.000000			
TTK	0.168	1.183 (1.002-1.396)	0.048	0.000000			
UTP3	-0.456	0.634 (0.455-0.884)	0.007	-0.372656	-0.516	0.597 (0.408-0.875)	**0.008**
ZMYM2	0.263	1.301 (1.028-1.647)	0.029	0.000000			

LASSO, least absolute shrinkage and selection operator; Coef, coefficient; Bold values indicates P < 0.05.

### The LLPS-Related Gene Signature Was Validated in the Test Set

**Figure 3:**
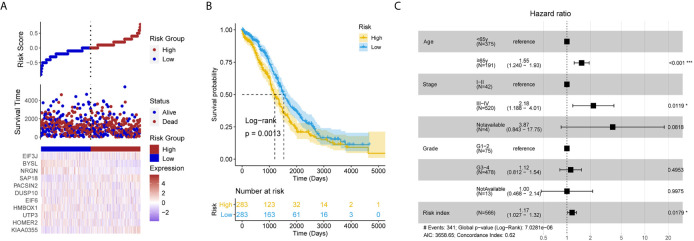
The liquid-liquid phase separation-related gene signature was validated in the test set. **(A)** The risk plot of test set based the 11 liquid-liquid phase separation-related gene signature. **(B)** The patients with high risk index had significantly shorter overall survival than those with low risk index in the test set. **(C)** The risk index remains significant compared routine clinical features.

After removing the TCGA-OV patients without survival information, and a total of 566 patients remained in the test set (**Table S1**). Each individual in the test set was also assigned a RI according to the formula (**Figure 3A**). It is exciting that the RI remained associated with poor prognosis (HR = 1.211, 95% CI for HR = 1.070–1.372, P = 0.003). It also successfully divided patients into high- and low-risk groups, the patients with high RI had significantly shorter OS than those with low RI (**Figure 3B**). Moreover, the LLPS-related gene signature RI is an independent prognostic factor adjusted by some clinical features (**Figure 3C**).

### Pathways Involved in These 11 Candidate Genes

**Figure 4:**
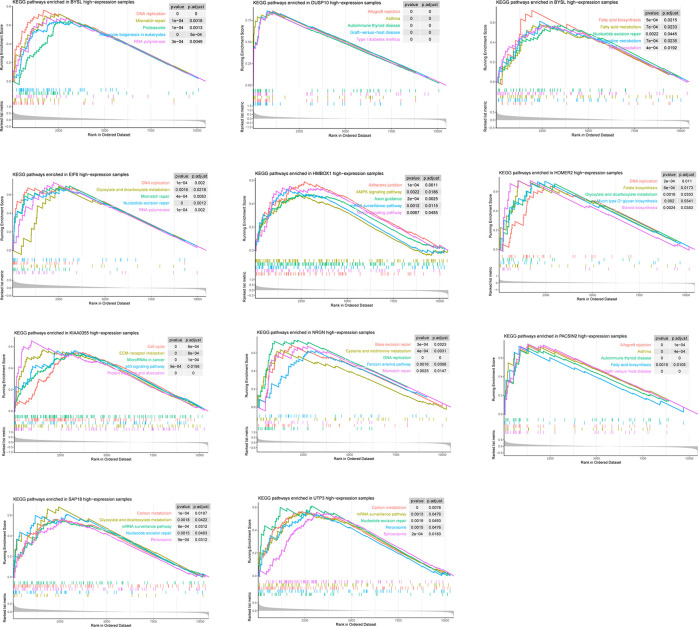
Pathways involved in these 11 candidate genes.

According to the GSEA results, the 11 candidate genes may involve in various pathways (**Figure 4**). For instance, the high expression of BYSL may associate with activation of DNA replication, Mismatch repair, and Proteasome. However, LLPS is the introduction of physical and chemical concepts to explain biological phenomena, the specific link between these pathways and LLPS remains to be further elucidated.

## Discussion

LLPS provides a new framework to understand and interpret cancer, with potentially new way for treatment. The mutation of LLPS-related gene may lead to aberrant forms of LLPS (26–28), the aberrant forms of LLPS contribute to the abnormal activity in cancer-related pathways (29). In the present study, we found that differentially expressed LLPS-related genes in EOC were involved in multiple cancer-related pathways, such as MAPK signaling pathway, cell cycle, and DNA replication. This indicated that LLPS in EOC was complicated. We also found that the expression patterns of LLPS-related genes were associated with prognosis in EOC and proposed an LLPS-related gene signature for predicting prognosis. Our LLPS-related gene signature was constructed and validated in two independent data sets based on different platforms. Thus, this might indicate that this LLPS-related gene signature is still robust in different populations and suitable for different platforms. In some previous studies (30, 31), univariate and multivariate Cox regression analyses were used but lack of LASSO analysis to create the prognostic gene signatures. However, these previous studies may encounter overfitting problems and not validated in independent data sets. The LASSO method was used for the optimal selection of features in high-dimensional data with a robust predictive value and low correlation between each other to prevent overfitting (32). Thus, our LLPS-related gene signature was validated in independent data sets. Moreover, the LLPS-related gene signature is independent prognostic factor compared clinical features, including age, pathological staging, and grade. According to the risk score system, the patients at high risk may be followed up more frequently and accept more active management than those at low risk.

The present LLPS-related gene signature consists of 11 LLPS-related genes. Unsurprisingly, some of the 11 LLPS-related genes were reported associated with EOC, such as a previous study proposed that altered EIF6 expression is associated with clinicopathological features in EOC (33), and low expression level of HMBOX1 in EOC may accelerate cell proliferation by inhibiting cell apoptosis (34). BYSL may be an oncogene in various cancer, including hepatocellular carcinoma (35), glioblastoma (36), and diffuse large B-cell lymphoma (37). NRGN was reported as a tumor suppressor in glioma cells (38), and we found that it is also associated with good prognosis in EOC. SAP18 was reportedly associated with the promotion of cell invasion and angiogenesis in virus oncogenic (39). PACSIN2 polymorphism was associated with thiopurine metabolism in children with acute lymphoblastic leukemia (40). The role of DUSP10 in cancer may be related to specific cancer types, some studies indicated that it is an oncogene, while in other studies indicated that it is a tumor suppressor (41). Although further molecular experiments are required, our GSEA results may help reveal the biological functions of these 11 candidate genes in EOC.

Although the present study may provide new insight into the risk stratification in EOC, several limitations should be noticed. First, the LLPS-related gene signature was proposed through retrospective study, prospective study is needed before it is used in clinical practice. Second, molecular function experiments were lacking in our present study, thus, it is not clear whether these genes are causal or merely prognostic markers in EOC.

In conclusion, we found significantly different expression patterns of LLPS-related genes in EOC compared to normal ovarian surface epithelium, and constructed and validated an LLPS-related gene signature as a prognosis tool in EOC through integrated analysis of multiple data sets.

## Data Availability Statement

Publicly available data sets were analyzed in this study. These data can be found here: https://www.ncbi.nlm.nih.gov/geo/, https://portal.gdc.cancer.gov/.

## Author Contributions

YQ designed the study. YQ analyzed the data and wrote the manuscript. MP and XC participated in analysis and interpretation of the data and reviewed the article. All authors contributed to the article and approved the submitted version.

## Funding

This work was supported by Science and Technology Plan Project of Maoming (grant number: 2019369) and High-level Hospital Construction Research Project of Maoming People’s Hospital.

## Conflict of Interest

The authors declare that the research was conducted in the absence of any commercial or financial relationships that could be construed as a potential conflict of interest.

## Supplementary Material

Supplementary Figure 1 The differentially expressed liquid-liquid phase separation-related genes in epithelial ovarian cancer. **(A)** The vocano plot of the liquid-liquid phase separation-related genes. Red represents up-regulated genes, blue represents down-regulated genes, and grey represents not significantly differential expression. **(B)** The expression heatmap of the differentially expressed liquid-liquid phase separation-related genes.

Supplementary Figure 2 Gene ontology terms and pathways involved in differentially expressed liquid-liquid phase separation-related genes. **(A)** cellular component, **(B)** biological process, **(C)** molecular function, and **(D)** Kyoto Encyclopedia of Genes and Genomes pathways. Log2FC, log2 (fold change).
