# Supplementary figures and images for: A Liquid-Liquid Phase Separation-Related Gene Signature as Prognostic Biomarker for Epithelial Ovarian Cancer
Source: Front Oncol. 2021 Jun 8;11:671892. doi: 10.3389/fonc.2021.671892 (PMC8217755; doi:10.3389/fonc.2021.671892)

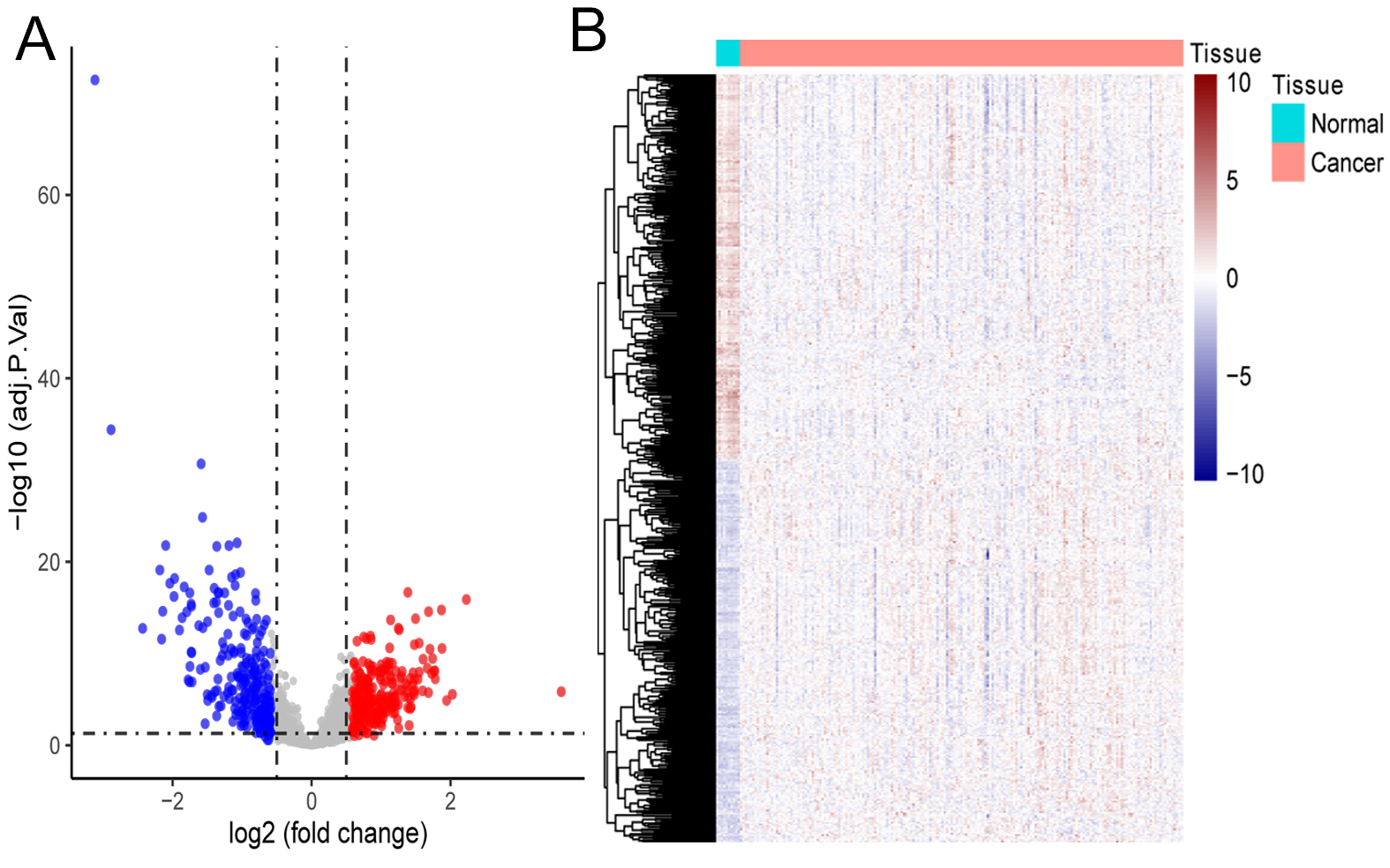

Supplement: Supplementary Figure 1 — The differentially expressed liquid-liquid phase separation-related genes in epithelial ovarian cancer. (A) The vocano plot of the liquid-liquid phase separation-related genes. Red represents up-regulated genes, blue represents down-regulated genes, and grey represents not significantly differential expression. (B) The expression heatmap of the differentially expressed liquid-liquid phase separation-related genes. [file Image_1.jpeg]

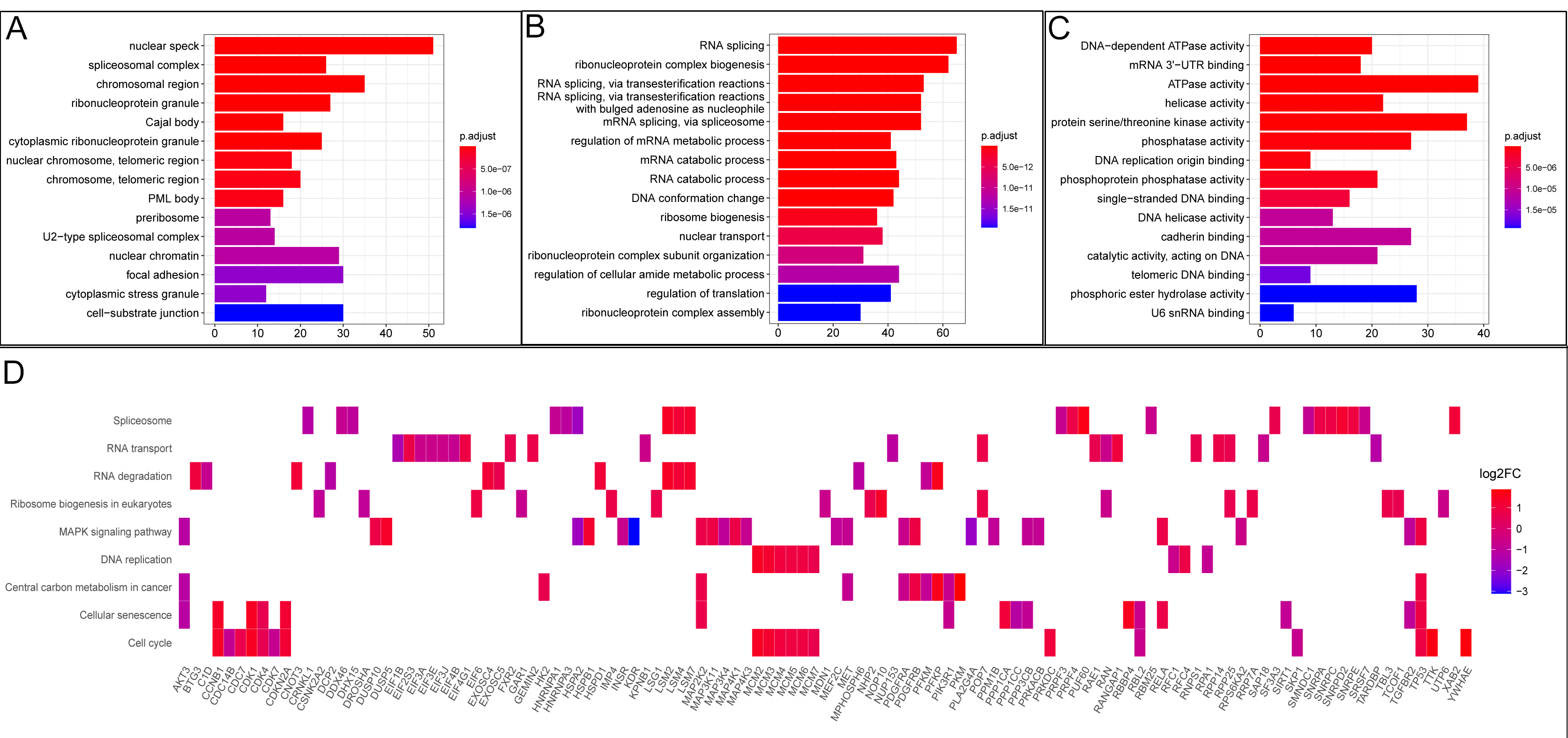

Supplement: Supplementary Figure 2 — Gene ontology terms and pathways involved in differentially expressed liquid-liquid phase separation-related genes. (A) cellular component, (B) biological process, (C) molecular function, and (D) Kyoto Encyclopedia of Genes and Genomes pathways. Log2FC, log2 (fold change). [file Image_2.jpeg]
